# Characteristics and therapeutic profile of TBI patients who underwent bilateral decompressive craniectomy: experience with 151 cases

**DOI:** 10.1186/s13049-022-01046-w

**Published:** 2022-11-17

**Authors:** Jian-Lan Zhao, Jie Song, Qiang Yuan, Yi-Feng Bao, Yi-Rui Sun, Zhi-Qi Li, Cai-Hua Xi, Hai-Jun Yao, Mei-Hua Wang, Gang Wu, Zhuo-Ying Du, Jin Hu, Jian Yu

**Affiliations:** 1 Department of Neurosurgery, National Center for Neurological Disorders, Neurosurgical Institute of Fudan University, Shanghai Clinical Medical Center of Neurosurgery, Shanghai Key Laboratory of Brain Function and Restoration and Neural Regeneration, Huashan Hospital, Fudan University, 12 Wulumuqi Zhong Road, Shanghai, 200040 China; 2 Department of Neurosurgery and Neurocritical Care, Huashan Hospital, Fudan University, Shanghai, 200040 China

**Keywords:** Traumatic brain injury, Bilateral decompressive craniectomy, Intracranial pressure monitoring, Unplanned secondary surgery, Outcome

## Abstract

**Background:**

Decompressive craniectomy (DC) and intracranial pressure (ICP) monitoring are common approaches to reduce the death rate of Traumatic brain injury (TBI) patients, but the outcomes of these patients are unfavorable, particularly those who receive bilateral DC. The authors discuss their experience using ICP and other potential methods to improve the outcomes of TBI patients who receive bilateral DC.

**Methods:**

Data from TBI patients receiving bilateral DC from Jan. 2008 to Jan. 2022 were collected via a retrospective chart review. Included patients who received unplanned contralateral DC after initial surgery were identified as unplanned secondary surgery (USS) patients. Patients’ demographics and baseline medical status; pre-, intra-, and postoperative events; and follow-up visit outcome data were analyzed.

**Results:**

A total of 151 TBI patients were included. Patients who underwent USS experienced more severe outcomes as assessed using the 3-month modified Rankin Scale score (*P* = 0.024). In bilateral DC TBI patients, USS were associated with worsen outcomes, moreover, ICP monitoring was able to lower their death rate and was associated with a lower USS incidence. In USS patients, ICP monitoring was not associated with improved outcomes but was able to lower their mortality rate (2/19, 10.5%, vs. 10/25, 40.0%; *P* = 0.042).

**Conclusion:**

The avoidance of USS may be associated with improved outcomes of TBI patients who underwent bilateral DC. ICP monitoring was a potential approach to lower USS rate in TBI patients, but its specific benefits were uncertain.

**Supplementary Information:**

The online version contains supplementary material available at 10.1186/s13049-022-01046-w.

## Background

An estimated 53–69 million individuals worldwide sustain a traumatic brain injury (TBI) annually [1], making it an international public health concern. Up to 2% of the population worldwide lives with neurological disabilities caused by TBIs [2, 3], and TBI remains the leading cause of death and disability [4]. Despite current medical treatment, intracranial hypertension can be induced by traumatic intracranial lesions or cerebral edema [5]. To avoid the potential cerebral herniation and other complications induced by intracranial hypertension, decompressive craniectomy (DC) could be selected [6, 7], but whether it is able to benefit TBI patients is debate.

Even with surgical evacuation of the injured brain and hematoma, the outcomes of TBI patients remain unfavorable [8], particularly those of TBI patients who undergo bilateral frontotemporal DC surgeries [9]. Among these TBI patients, those who receive unplanned secondary surgeries (USSs) to achieve contralateral DC exhibited more severe outcomes [10]. To the best of our knowledge, no specific treatment is able to improve the outcomes of TBI patients after USS for contralateral DC. It seems that the best intervention would be to lower the incidence of USSs after a unilateral DC, but related studies are limited.

Intracranial pressure (ICP) monitoring is also could be considered in the treatment of TBI patients [7], but because of its higher cost, uncertain improvement in outcomes and potential adverse events [11-13], the routine use of ICP monitoring in TBI patients is still controversial. Whether the implantation of an ICP probe could benefit TBI patients, particularly those who underwent USSs for contralateral DC, is unclear.

Reports are limited on treating TBI patients who underwent bilateral DC. Here we outline our experience with 151 TBI patients who received bilateral DC. We evaluated whether ICP monitoring was associated with improved outcomes of these patients and in addition, whether ICP monitoring could reduce the occurrence of USSs for contralateral DC in TBI patients.

## Patients and methods

### Study design and patients

Patient data for 151 consecutive TBI who received bilateral DC from Jan. 2008 to Jan. 2022 were prospectively in a database and retrospectively reviewed. This study was approved by the ethic committee of Huashan Hospital Fudan University. Patients with TBI who were admitted to the Department of the Neurosurgery Neurotrauma Center at Huashan Hospital Fudan University were included. Informed consent was obtained from all individual participants. If a patient could not sign informed consent by himself/herself, such as patients with a Glasgow Outcome Scale (GOS) score of 1, informed consent was signed by his/her statutory guardian.

**Fig. 1:**
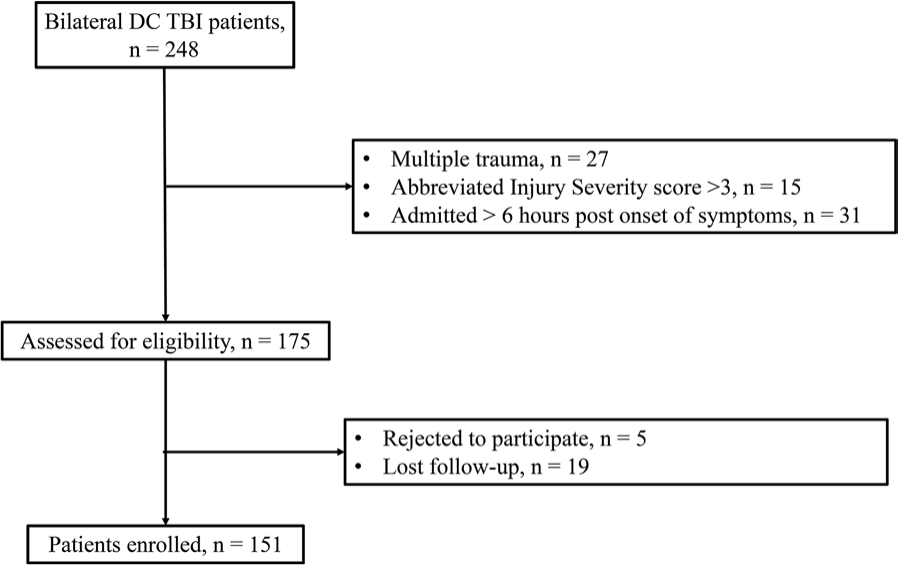
Flow chart of the study

Inclusion criteria are as follows. First, participants had to be computed tomography (CT) scan confirmed patients with TBI. CT signs of TBI included epidural hematoma, subdural hematoma (SDH), intraparenchymal hemorrhage (IPH), brain contusion, and brain laceration. Second, patients had to be > 18 years of age. Third, patients received bilateral frontal-temporal-parietal DC at discharging. Finally, participants had to be admitted within 6 h after injury. Patients with TBI with traumatic injury to a body region other than the brain with an Abbreviated Injury Severity score > 3, with penetrating brain injury and those already received unilateral DC in other hospitals were excluded. However, if a patient only received ICP monitoring implanted in other hospitals at admission, the patient would be enrolled (Fig. 1).

### Demographic data collection

Baseline characteristics, including age, sex, mechanism of injury, pupillary reaction to light, GCS score at admission, and type of injury, were recorded for all patients. Injury types were assessed based on initial CT scan on admission. Coagulopathy was defined as regular coagulation test results meeting one or more of the following criteria: platelet counts (PLT) < 100 × 109/L, international normalized ratio (INR) > 1.25, prothrombin time (PT) > 14 s, or activated partial thromboplastin time (APTT) > 36 s as our previously studies [14-16]. Peripheral blood analysis was performed for all patients within 6 h of injury at the Central Clinical Laboratory in Huashan Hospital.

### Operation procedures

The frontal-temporal DC was performed as previously reported to provide maximal decompression [5]. The area of bone flaps was 12*15 cm^2^ was performed as Guidelines for the Management of Severe Traumatic Brain Injury [7, 17]. If bilateral DC were performed, a strip of midline bone bridge covering the superior sagittal sinus would be left [5]. During the DC process, intracerebral lesions, including hematoma, contused or lacerated brain tissue and et al. were removed as totally as possible, in addition, de-stretching duroplasty was applied. ICP probe and intraventricular drainage system were implanted into lateral ventricle as previously reported. For indications of ICP implantation varied in the duration of this retrospective research, whether ICP monitoring was applied was decided by neurosurgeons who performed DC. If ICP probes were applied, it was preformed accompanied with or prior to 1st DC, and no ICP iprobes were implanted between initial DC and unplanned contralateral DC.

### Unplanned secondary surgery patients

According to different operation plans. we divided all bilateral DC patients into four categories: (1) *Pre-operative scheduled bilateral DC*: bilateral lesions were detected at admission. (2) *Intraoperative scheduled contralateral DC*: Unilateral lesions were identified at admission and received unilateral DC. But according to intraoperative ICP values, abnormal brain swelling and et al, these patients were suspected to suffer contralateral lesions. After confirmed by intraoperative CT scans, these patients received immediately contralateral DC. (3) *Post-operative immediately contralateral DC* patients: Unilateral lesions were identified at admission, and after initial DC, no abnormal signs, symptoms or intraoperative CT scans findings were detected during operations. Abnormal high ICP value (> 20 mmHg) were detected before these patients were sent back to NICU. These patients received immediately CT scans and contralateral lesions were detected, and then these patients were sent back to operation rooms without delay to receive contralateral DC. (4) *Unplanned contralateral DC* patients: These patients were safely sent back to NICU after unilateral DC. Contralateral lesions were identified and contralateral DC were performed during hospitalization. These patients were identified as unplanned secondary surgery (USS) patients in this study.

### Treatment protocol

The patients were treated in accord with the latest guidelines, but detailed therapeutic approaches were determined by neurosurgeons who performed operations. If ICP implantation were applied, the cerebral perfusion pressure (CPP) was maintained at 75–90 mm Hg at all times by keeping the mean arterial pressure at 90–100 mm Hg and the ICP at < 20 mm Hg. Systolic blood pressure was required to be maintained under 140 mmHg. Corticosteroids were not used. Body temperature, respiratory rate, heart rate, blood pressure, cardiac rhythm, and oxygen saturation were monitored continuously. Serum glucose, blood gas, and serum electrolyte values were measured regularly and kept within normal range. Intraoperative CT scans were performed before operations ending or when abnormal brain swelling, abnormal ICP values and et al. occurred. Postoperative CT scans were routinely performed 24 h, 72 h and 7 days after operations or when neurological deficits occurred. All patients were evaluated and treated by full-time neurosurgeons with specific training in critical care.

### Assessments

A specialist in physical medicine and rehabilitation determined the neurological outcome at 3 months after injury. The primary outcome of patients with TBI was assessed using the mortality (Grade 6) and functional outcome at 90 days after admission by using the modified Rankin scale (mRS) as our previous studies [14], through outpatient interviews or over the telephone. mRS score of 4–6 was considered a poor outcome, and mRS score of 0–3 was considered a good outcome. The rates of operation, length of ICU stay and the rate of serious adverse events, including kidney dysfunction, brain infarction and et al., were collected at discharging.

### Statistical analysis

Continuous variables were expressed as mean ± standard deviation (SD) or median (interquartile range), and categorical variables were expressed as percentages. The univariate analyses of categorical data were performed using the χ^2^ test. Equality of variance was assessed using the Levene test. Normally distributed variables were compared using Student t test or one-way analysis of variance, whereas nonnormally distributed variables were compared using the Kruskal–Wallis or Mann–Whitney U test.

After the univariate analyses, a forward stepwise logistic regression analysis of the 3-month outcome was used to develop the prediction models and adjust for multiple predictors of 3-month outcome. All statistical tests were 2-tailed, and *P* < 0.05 was considered statistically significant. Statistical analysis was carried out using SPSS 23.0 (IBM, Armonk, New York, USA) and MedCalc statistical software (version 15.2.2, MedCalc Software bvba, Ostend, Belgium).

## Results

Patients who received care between Jan. 2008 to Jan. 2022 were initially enrolled, with the last follow-up visit occurring in April 2022. Of 208 eligible patients, 57 (27.4%) were excluded before the analysis. The 151 included patients were first dichotomized according to their 3-month modified Rankin Scale (mRS) score, including whether they suffered mortality (mRS grade 6). With regard to all baseline characteristics (Table 1), the two groups were similar at baseline apart from age, pupillary reactions, Glasgow Coma Scale (GCS) score, white blood cell (WBC) count, and the neutrophil-lymphocyte ratio (NLR) (*P* < 0.05). More USS patients were found in the poor-outcome group (*P* = 0.016). The outcomes of TBI patients who underwent bilateral DC were similar regardless of whether ICP monitoring was applied (*P* = 0.091); however, higher ICP values were detected in the poor-outcome group (*P* < 0.001).

**Table 1:** Baseline Characteristics of all Bilateral DC TBI patients according to 3-Month Outcome

	Poor outcome (mRS 4–5, and death)	Good outcome (mRS 0–3)	*P* value
n	94	57	151
Age (years)	54.13 (24.14–63.21)	51.18 (22.13–58.93)	< 0.001*
Male (n, %)	71 (75.5)	41 (71.9)	0.702
Mechanism of injury (n, %)			0.115
Motor vehicle accident	33 (35.1)	11 (19.3)	
Fall	13 (13.8)	14 (24.6)	
Stumble	19 (20.2)	15 (26.3)	
Blow to head	17 (18.1)	13 (22.8)	
Others	12 (12.7)	4 (7.1)	
Pupillary reactions (n, %)			< 0.001*
Both reacting	7 (7.4)	39 (68.4)	
One reacting	43 (45.8)	17 (29.8)	
None reacting	44 (46.8)	1 (1.8)	
GCS at admission	6.32 ± 3.16	12.19 ± 2.08	< 0.001*
Type of injury (n, %)^#^			
SDH	34 (36.1)	18 (31.6)	0.601
EDH	16 (17.02)	12 (21.05)	0.663
IPH	26 (27.6)	24 (42.11)	0.076
tSAH	42 (44.7)	37 (64.9)	0.019*
DAI	22 (23.4)	14 (24.6)	0.999
Skull fracture	27 (28.7)	22 (28,5)	0.999
Coagulopathy	36 (38.3)	8 (14.04)	0.0016*
WBCs, (x10^9^/L)	19.91 ± 6.17	11.38 ± 6.27	< 0.001*
NLR, (%)	28.11 ± 14.52	8.18 ± 5.14	< 0.001*
USS	34 (36.2)	10 (17.5)	0.016*
ICP monitoring	51 (54.3)	39 (68.4)	0.091
ICP value (mmHg)	14.78 (10.13–19.93)	12.12 (8.34–16.41)	< 0.001*

Data are given as mean ± SD, n (%), or median (IQR) unless otherwise noted

*DAI* diffused axonal injury, *EDH* extradural hematoma, *ICP* intracranial pressure, *IPH* intra-parenchyma hematoma, *NLR* neutrophil-to-lymphocyte ratio, *SDH* subdural hematoma, *tSAH* traumatic subarachnoid hemorrhage, *USS* unplanned secondary surgery, *WBC* white blood cells

*P < 0.05

^#^Patients may suffer more than one item

We then performed multivariate logistic regression to evaluate the independent risk factors for the 3-month outcome. As shown in Table 2, the significant independent variables included age, GCS score at admission, coagulopathy, USS, and the NLR (*P* < 0.05). These results suggest that worse outcomes of TBI patients who underwent bilateral DC may be associated with USS.

**Table 2:** Multivariate logistic regression analysis predicting the 3-month outcome

Independent variable	Adjusted OR (95% CI)	*P* value
Age	1.04 (1.01–1.06)	< 0.001
GCS	0.67 (0.58–0.77)	< 0.001
Pupillary reactions	0.87 (0.46–0.94)	0.032
Coagulopathy	1.58 (1.18–1.82)	< 0.001
USS	2.76 (1.55–3.94)	< 0.001
NLR	1.08 (1.01–1.29)	0.028

*ICP* intracranial pressure, *NLR* neutrophil-to-lymphocyte ratio, *USS* unplanned secondary surgery

**Fig. 2:**
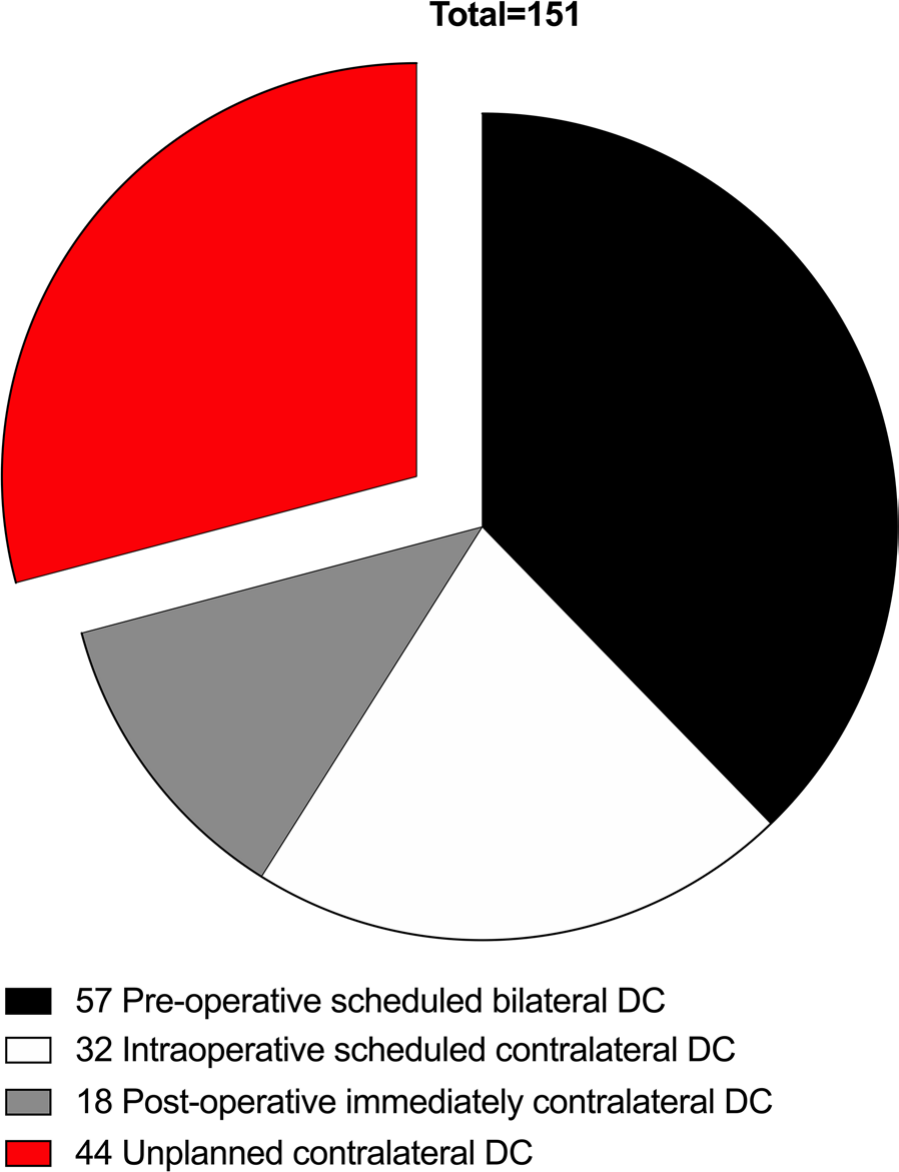
Patient characteristics according to bilateral DC operation plans. *Pre-operative scheduled bilateral DC* patients: bilateral lesions were detected at admission. *Intraoperative scheduled contralateral DC* patients: Unilateral lesions were identified at admission and received unilateral DC. But according to intraoperative ICP values, abnormal brain swelling and et al. these patients were suspected to suffer contralateral lesions. After confirmed by intraoperative CT scans, these patients received immediately contralateral DC. *Post-operative immediately contralateral DC* patients: Unilateral lesions were identified at admission, and after initial DC, no abnormal signs, symptoms or intraoperative CT scans findings were detected during operations. Abnormal high ICP value (> 20 mmHg) were detected before these patients were sent back to NICU. These patients received immediately CT scans and contralateral lesions were detected, and then these patients were sent back to operation rooms without delay to receive contralateral DC. *Unplanned contralateral DC* patients: These patients were safely sent back to NICU after unilateral DC. Contralateral lesions were identified and contralateral DC were performed during hospitalization

**Fig. 3:**
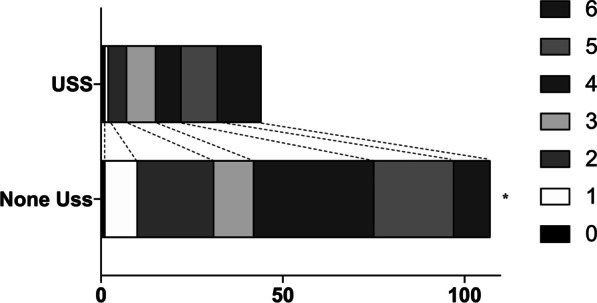
The outcomes of TBI patients who underwent bilateral DC were significantly aggravated by USS, according to their 3-month mRS scores. *P < 0.05

Among the 151 bilateral DC patients, bilateral lesions were detected in 57 patients at admission, and the 57 patients received pre-operative scheduled bilateral DC. Unilateral lesions were identified in 94 patients at admission, and 32 of the 94 patients received intraoperative scheduled contralateral DC. Moreover, 18 of the 94 patients were categorized as “Post-operative immediately contralateral DC”. Lastly, the rest 44 of the 94 patients received unplanned contralateral DC (Fig. 2). We evaluated the outcomes between the included patients who received USS (44/151, 29.17%) and those who did not (107/151, 70.86%) by assessing their 3-month mRS score. It was indicated that USS was associated with worsen functional outcomes (*P* = 0.024, Fig. 3).

We furtherly analyzed characteristics of included patients who received USS or not (Table 3). It was suggested that motor vehicle accidents were more common in USS patients, and that the differences of pupillary reactions were significantly between patients suffered USS or not (*P* < 0.05). Moreover, coagulopathy may be associated with USS (*P* < 0.05). However, by performing multivariate logistic analysis, no variable was independently associated with USS (Additional file 1: Table S1). In addition, the incidence of hydrocephalus at 3-month was higher in USS patients (18/44, 40.9%, vs. 22/107, 20.6%; *P* = 0.0145). Similarly, more USS patients suffered postoperative seizures compared with non-USS patients (16/44, 36.3%, vs. 14/107, 13.1%; *P* = 0.0028, Additional file 2: Table S2).

**Table 3:** Baseline Characteristics of bilateral DC TBI patients according to receive USS or not

	USS, n = 44	No USS, n = 107	*P* value
Age (years)	48.13 (23.17–56.39)	44.18 (21.13–61.24)	0.767
Male (n, %)	27 (61.3)	95 (88.7)	< 0.001*
Mechanism of injury (n, %)			0.02*
Motor vehicle accident	21 (47.7)	23 (21.5)	0.0013*
Fall	8 (18.2)	19 (17.7)	
Stumble	7 (15.9)	27 (25.2)	
Blow to head	5 (11.3)	25 (23.4)	
Others	3 (6.8)	13 (12.1)	
Pupillary reactions (n, %)			< 0.001*
Both reacting	10 (22.7)	36 (33.6)	
One reacting	31 (70.5)	29 (27.1)	
None reacting	3 (6.8)	42 (39.3)	
GCS at admission	10.57 ± 2.25	11.32 ± 3.12	0.619
Initial Type of injury (n, %)^#^			
SDH	12 (27.3)	40 (37.4)	0.263
EDH	5 (11.3)	23 (21.5)	0.172
IPH	6 (13.6)	34 (31.7)	0.025*
tSAH	18 (40.9)	61 (58.65)	0.071
DAI	8 (18.2)	28 (26.1)	0.409
Skull fracture	16 (36.4)	41 (42.3)	0.580
Coagulopathy	19 (43.2)	25 (23.1)	0.018
WBCs, (x10^9^/L)	14.53 ± 4.93	12.14 ± 6.13	0.176
NLR, (%)	10.71 ± 5.52	13.01 ± 8.26	0.239
ICP value (mmHg)	14.78 (10.13–19.93)	12.12 (8.34–16.41)	0.079

Data are given as mean ± SD, n (%), or median (IQR) unless otherwise noted

*DAI* diffused axonal injury, *EDH* extradural hematoma, *ICP* intracranial pressure, *IPH* intra-parenchyma hematoma, *NLR* neutrophil-to-lymphocyte ratio, *SDH* subdural hematoma, *tSAH* traumatic subarachnoid hemorrhage, *USS* unplanned secondary surgery, *WBC* white blood cells

*P < 0.05.

^#^Patients may suffer more than one item

**Fig. 4:**
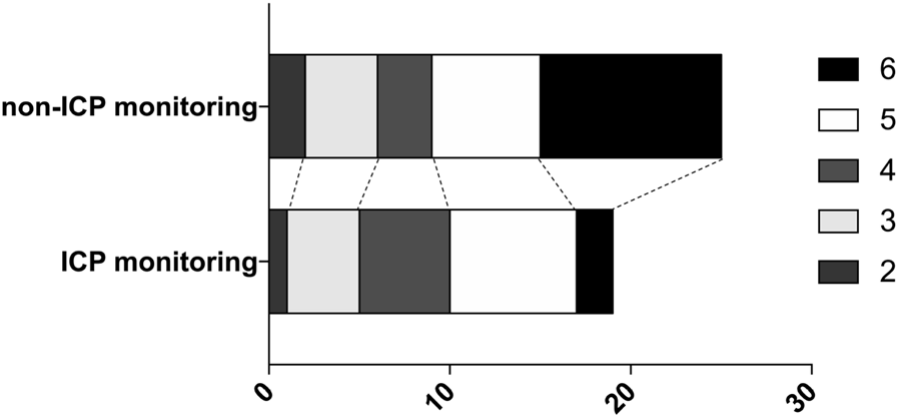
The outcomes of TBI patients who underwent USS could be improved via ICP monitoring, according to their 3-month mRS scores

To evaluate effects of ICP monitoring on the outcomes of TBI patients who underwent bilateral DC, we divided all included patients into two groups according to whether ICP monitoring was performed. As shown in Table 4, significant differences were detected for WBC count (*P* < 0.05). Then, we analyzed the details of the 3-month outcomes between patients who did or did not received ICP monitoring and evaluated the safety of ICP monitoring (Table 5). ICP monitoring was associated with a lower incidence of USS (*P* = 0.011) and mortality (*P* = 0.0104), although the 3-month mRS scores were similar between groups (*P* = 0.448). The length of stay in the intensive care unit and occurrence rate of adverse events and complications, including progressive intracranial hemorrhage and hydrocephalus, were not significantly different between the two groups (*P* > 0.05). Finally, among the 44 USS patients, ICP monitoring was performed on 19 patients. Based on the 3-month mRS score, we found that ICP monitoring was not associate with improved outcomes of USS patients (*P* = 0.237, Fig. 4), but was able to lower their death rate (2/19, 10.5%, vs. 10/25, 40.0%; *P* = 0.042).

**Table 4:** Patients’ characteristics according to ICP monitoring or not

	ICP monitoring, n = 90	None-ICP monitoring, n = 61	*P* value
Age (yrs)	45.38 (31.7–58.4)	48.21 (29.14–57.13)	0.661
Male (n, %)	63 (70.0)	41(67.3)	0.725
Mechanism of injury (n, %)			0.635
Motor vehicle accident	26 (28.7)	18 (29.5)	
Fall	20 (22.2)	7 (11.4)	
Stumble	22 (24.4)	12 (19.7)	
Blow to head	22 (24.4)	8 (8.8)	
Others	10 (11.1)	6 (6.6)	
Pupillary reactions (n, %)			0.719
Both reacting	26 (28.9)	20 (32.8)	
One reacting	35 (38.9)	25 (40.9)	
None reacting	29 (32.2)	16 (26.3)	
GCS at admission	9.37 ± 4.96	10.59 ± 3.88	0.778
Type of injury (n, %)^#^			
SDH	33 (36.7)	19 (31.2)	0.601
EDH	17 (18.9)	11 (18.1)	0.999
IPH	34 (37.8)	16 (26.2)	0.161
tSAH	47 (52.2)	32 (52.5)	0.999
DAI	21 (23.3)	15 (24.6)	0.848
Skull fracture	34 (37.8)	23 (37.7)	0.999
Coagulopathy (n, %)	18 (20.0)	12 (19.6)	> 0.999
WBCs, (x10^9^/L)	16.09 ± 5.17	12.31 ± 3.15	< 0.001*
NLR, (%)	12.14 ± 9.52	11.48 ± 5.54	0.431

Data are given as mean ± SD, n (%), or median (IQR) unless otherwise noted

*DAI* diffused axonal injury, *EDH* extradural hematoma, *ICP* intracranial pressure, *IPH* intra-parenchyma hematoma, *NLR* neutrophil-to-lymphocyte ratio, *SDH* subdural hematoma, *tSAH* traumatic subarachnoid hemorrhage, *USS* unplanned secondary surgery, *WBC* white blood cells

*P < 0.05.

^#^Patients may suffer more than one item

**Table 5:** 3-Month outcomes and safety of ICP monitoring

	ICP monitoring, n = 90	None-ICP monitoring, n = 61	*P* value
Progressive intracranial hemorrhage (n, %)			
Hematoma enlargement	13 (14.4)	6 (9.8)	0.462
New hemorrhagic lesion	7 (7.8)	4 (6.6)	0.999
Operations plans			
Pre-operative scheduled bilateral DC	33 (36.7)	24 (39.3)	0.864
Intraoperative scheduled contralateral DC	20 (22.2)	12 (19.8)	0.839
Post-operative immediately contralateral DC	18 (20.0)	0 (0)	< 0.001
USS, (n, %)	19 (21.1)	25 (40.9)	0.011*
Length of ICU stay	9 (6–17)	13 (8–22)	0.057
Complications and adverse events (n, %)^#^			
Hydrocephalus	15 (16.7)	13 (21.3)	0.525
CNS Infection	6 (6.7)	3 (4.9)	0.740
Others	12 (13.3)	6 (9.8)	0.213
Modified Rankin Scale at 90 days (n, %)			0.448
0: No symptoms at all	1 (1.1)	1 (1.6)	
1: No substantive disability despite symptoms	5 (5.5)	1 (1.6)	
2: Slight disability	7 (7.6)	4 (6.5)	
3: Moderate disability requiring some help	22 (24.1)	3 (4.9)	
4: Moderate–severe disability requiring assistance with daily living	28 (30.7)	16 (26.4)	
5: Severe disability, bed­bound and incontinent	19 (20.2)	21 (34.4)	
6: Death by 90 days (mortality)	8 (8.8)	15 (24.6)	0.0104*

Data are given as n (%), or median (IQR) unless otherwise noted

*DC* decompressive craniectomy, *ICP* intracranial pressure, *ICU* intensive caring unites, *IPH* intra-parenchyma hematoma, *NLR* neutrophil-to-lymphocyte ratio, *SDH* subdural hematoma, *tSAH* traumatic subarachnoid hemorrhage, *USS* unplanned secondary surgery, *WBC* white blood cells

*P < 0.05

^#^Patients may suffer more than one item

## Discussion

Based on our therapeutic experience of the 151 TBI patients who received bilateral DC, we found that post-operative immediately contralateral DC improve outcomes of TBI patients who undergo bilateral DC compared to secondary surgery. ICP monitoring could increase the number of post-operatives immediately contralateral DC, and hence improve outcome (Table 5).

### Potential therapeutic approach to improve unfavorable outcomes in TBI patients who undergo bilateral DC: USS for contralateral DC should be avoided

The outcomes of TBI patients who receive bilateral DC are poor [5], but the specific pathophysiological process of this acute traumatic neurological disease is not known; thus, the most favorable therapeutic approach is unclear. In other studies [9, 10], the outcomes of TBI patients who underwent bilateral DC were also unsatisfactory. In our study, we found that TBI patients who underwent unplanned contralateral DC surgeries experienced more severe outcomes compared with those who received bilateral DC in one operation. To the best of our knowledge, this is the first study to demonstrate that preventing unplanned contralateral DC surgery may improve the outcomes of TBI patients who undergo bilateral DC.

USS, or unplanned reoperation, can be considered a type of severe operation-related adverse event [18], because if non-operative treatments were used successfully in patients, no USS would be needed. An unplanned return to the operating room is associated with an 11.65-fold increase in the risk of hospital readmission, and the quality of life of patients who undergo USS is worse [19]. The outcomes of breast cancer patients were found to be aggravated after USS [20], with similar reports in patients with acute spinal cord injury [21] and cervical spondylitis myelopathy [22]. More severe complications can be induced by USS in patients with femur fractures and spinal cord injuries [23]. It is reasonable that USS should be avoided in any field.

In our study, the cause of unplanned contralateral DC surgery in TBI patients varied; contralateral brain contusion or subdural hematoma that was not detected before the initial operation was the most common cause. To avoid potential cerebral herniation induced by these undetected lesions, unplanned contralateral DC surgery will have to be performed. It has been previously reported that unilateral DC cannot improve the outcomes of TBI patients, but it does reduce the mortality rate [24, 25], and we also found that unplanned contralateral DC surgery could worsen the outcomes of these TBI patients. These results suggest that careful examination is necessary to find a potential contralateral brain contusion or subdural hematoma on admission or even during the initial operation to avoid USS. However, the specific mechanism by which USS exacerbates TBI patients’ outcomes remains unclear, and further studies are required.

### Should ICP monitoring be performed on TBI patients who undergo bilateral DC?

ICP monitoring is widely used in patients with TBI, intracerebral hemorrhage, subarachnoid hemorrhage, and other conditions, although there is no evidence that the outcomes of TBI patients could be significantly improved by ICP monitoring, although the level of evidence for ICP monitoring in TBI patients is IIB [7]. A meta-analysis indicated that the mortality rate of TBI patients decreased after ICP monitoring [26, 27], but most patients included in these studies received only unilateral DC, and whether TBI patients who undergo bilateral DC should receive ICP monitoring remains controversial.

In our study, we found that ICP monitoring could lower the incidence of USS in TBI patients, and its potential mechanism maybe that abnormal ICP values maybe an indicator of contralateral lesions. As shown in Fig. 2 and Table 4, within the category “Post-operative immediately contralateral DC”, the 18 patients all received ICP implantation. Their intraoperative signs, symptoms or CT scans were normal, but between the period after unilateral DC ending and before arrival to NICU, their abnormal high ICP value (> 18 mmHg) was detected and received immediately CT scans. If ICP were not applied, the 18 patients would have to receive USS. Actually, within the 19 USS patients who received ICP implantation, abnormal high ICP values were detected in 7 patients immediately after their first DC, and CT scans were performed on the 7 patients without any delay, but no significantly abnormal CT scan findings were detected. On the other hand, within the 25 USS patients who did not receive ICP implantation, although their intraoperative signs, symptoms or CT scans were normal before be sent back to NICU, some patients may suffer potential intracranial hypertension, unfortunately, we cannot detect this abnormal sign and finally USS occurred.

However, the outcomes of TBI patients who undergo bilateral DC cannot be improved through ICP monitoring, even though the USS rate can be reduced and the outcomes of USS patients could be alleviated by ICP monitoring. This finding was consistent with previous studies [28]. In TBI patients who undergo uni- or bilateral DC, other than the death rate, the patient outcome cannot be improved through ICP monitoring. Nevertheless, the lowering of the death rate may in turn result in an increase in the number of disabled patients, which increases the social, family, and financial burden. Further studies are required to elucidate the most favorable therapeutic approach for treating TBI patients based on information provided by ICP monitoring.

### Limitations

First, the nature history of TBI patients who received bilateral DC made our study considerable heterogeneity in patient population, and made it vulnerable to biases. Second, we tend to admit patients with more severe injuries, which could have caused admission bias. Therefore, caution should be exercised in interpreting our conclusions, and a prospective multi-center study is needed to further elucidate the potential mechanism underlying the process described here. In our further research, we will focus on whether ICP monitoring could benefit TBI patients with polytrauma and TBI patients within 24 h after onset of injury.

## Conclusion

To our best knowledge, this the largest available collection of outcomes for TBI patients who received bilateral DC. This is the largest retrospective series for TBI patients who received bilateral DC demonstrates that the outcomes of these TBI patients can be aggravated by unplanned contralateral DC surgery, moreover, ICP monitoring can reduce the incidence rate of USS and lower the death rate of USS TBI patients. We wish to share our relatively large experience and therapeutic approaches for TBI patients.

## Supplementary Information

**Additional file 1: Table S1.** Multivariate logistic regression analysis predicting USSs.

**Additional file 1: Table S2.** 3-month complications comparison between USS and none-USS patients.

## Data Availability

All data and materials are available from corresponding author, Jin Hu, on reasonable request.

## Abbreviations

CPP: Cerebral perfusion pressure
DAI: Diffused axonal injury
DC: Decompressive craniectomy
EDH: Extradural hematoma
ICP: Intracranial pressure monitoring
IPH: Intra-parenchyma hematoma
GCS: Glasgow coma scale
NLR: Neutrophil-to-lymphocyte ratio
mRS: Modified rankin scale
SDH: Subdural hematoma
TBI: Traumatic brain injury
tSAH: Traumatic subarachnoid hemorrhage
USS: Unplanned secondary surgery
WBC: White blood cells

## References

[1] Dewan MC, Rattani A, Gupta S, Baticulon RE, Hung YC, Punchak M (2018). Estimating the global incidence of traumatic brain injury. J Neurosurg..

[2] Hsia RY, Markowitz AJ, Lin F, Guo J, Madhok DY, Manley GT (2018). Ten-year trends in traumatic brain injury: a retrospective cohort study of California emergency department and hospital revisits and readmissions. BMJ Open.

[3] Gardner RC, Dams-O’Connor K, Morrissey MR, Manley GT (2018). Geriatric traumatic brain injury: epidemiology, outcomes, knowledge gaps, and future directions. J Neurotrauma.

[4] Jarrahi A, Braun M, Ahluwalia M, Gupta RV, Wilson M, Munie S (2020). Revisiting traumatic brain injury: from molecular mechanisms to therapeutic interventions. Biomedicines..

[5] Bao YH, Liang YM, Gao GY, Pan YH, Luo QZ, Jiang JY (2010). Bilateral decompressive craniectomy for patients with malignant diffuse brain swelling after severe traumatic brain injury: a 37-case study. J Neurotrauma.

[6] Stocchetti N, Maas AI (2014). Traumatic intracranial hypertension. N Engl J Med.

[7] Carney N, Totten AM, O’Reilly C, Ullman JS, Hawryluk GW, Bell MJ (2017). Guidelines for the management of severe traumatic brain injury, fourth edition. Neurosurgery.

[8] Barthelemy EJ, Melis M, Gordon E, Ullman JS, Germano IM (2016). Decompressive Craniectomy for Severe Traumatic Brain Injury: A Systematic Review. World Neurosurg.

[9] Walcott BP, Nahed BV, Sheth SA, Yanamadala V, Caracci JR, Asaad WF (2012). Bilateral hemicraniectomy in non-penetrating traumatic brain injury. J Neurotrauma.

[10] Choi YH, Lim TK, Lee SG (2017). Clinical features and outcomes of bilateral decompression surgery for immediate contralateral hematoma after craniectomy following acute subdural hematoma. Korean J Neurotrauma.

[11] Cardim D, Robba C, Schmidt B, Donnelly J, Schmidt EA, Bohdanowicz M (2019). Midline shift in patients with closed traumatic brain injury may be driven by cerebral perfusion pressure not intracranial pressure. J Neurosurg Sci.

[12] Melhem S, Shutter L, Kaynar A (2014). A trial of intracranial pressure monitoring in traumatic brain injury. Crit Care.

[13] Forsyth RJ, Raper J, Todhunter E (2015). Routine intracranial pressure monitoring in acute coma. Cochrane Database Syst Rev..

[14] Zhao JL, Du ZY, Sun YR, Yuan Q, Yu J, Wu X (2019). Intensive blood pressure control reduces the risk of progressive hemorrhage in patients with acute hypertensive intracerebral hemorrhage: a retrospective observational study. Clin Neurol Neurosurg.

[15] Zhao JL, Du ZY, Yuan Q, Yu J, Sun YR, Wu X (2019). Prognostic value of neutrophil-to-lymphocyte ratio in predicting the 6-month outcome of patients with traumatic brain injury: a retrospective study. World Neurosurg.

[16] Zhao JL, Lai ST, Du ZY, Xu J, Sun YR, Yuan Q (2020). Circulating neutrophil-to-lymphocyte ratio at admission predicts the long-term outcome in acute traumatic cervical spinal cord injury patients. BMC Musculoskelet Disord.

[17] Brain Trauma F, Neurological S, American Association of (2007). Congress of Neurological S. Guidelines for the management of severe traumatic brain injury. J Neurotrauma.

[18] Li Y, Helvie P, Farley FA, Abbott MD, Caird MS (2018). Complications after plate fixation of displaced pediatric midshaft clavicle fractures. J Pediatr Orthop.

[19] Pooshpas P, Lehman E, Aziz F (2018). Factors associated with increased risk of unplanned hospital readmission after endovascular aortoiliac interventions. Cureus.

[20] Jones SJ, Turton P, Achuthan R, Hogan BV, McKenzie SN, Kim B (2020). Analysis of secondary surgeries after immediate breast reconstruction for cancer compared with risk reduction. Plast Reconstr Surg Glob Open.

[21] Basu S, Solanki AM, Srivastava A, Shetty AP, Rajasekaran S, Jayaswal A (2020). Unplanned return to operation room (OR) following growing spinal constructs (GSCs) in early onset scoliosis (EOS)-a multi-centric study. Eur Spine J.

[22] Plano X, Ramirez M, Matamalas A, Haddad S, Garcia de Frutos A, Casamitjana JM (2019). 30-Day unplanned surgery in cervical spondylotic myelopathy surgically treated: a single-center experience. Eur Spine J.

[23] Perkins C, Buck JS, Karunakar MA (2019). Outcomes in the treatment of femur fractures in patients with pre-existing spinal cord injury. Bull Hosp Jt Dis.

[24] Timofeev I, Kirkpatrick PJ, Corteen E, Hiler M, Czosnyka M, Menon DK (2006). Decompressive craniectomy in traumatic brain injury: outcome following protocol-driven therapy. Acta Neurochir Suppl.

[25] Brown DA, Wijdicks EF (2017). Decompressive craniectomy in acute brain injury. Handb Clin Neurol.

[26] Yuan Q, Wu X, Sun Y, Yu J, Li Z, Du Z (2015). Impact of intracranial pressure monitoring on mortality in patients with traumatic brain injury: a systematic review and meta-analysis. J Neurosurg.

[27] Shen L, Wang Z, Su Z, Qiu S, Xu J, Zhou Y (2016). Effects of intracranial pressure monitoring on mortality in patients with severe traumatic brain injury: a meta-analysis. PLoS ONE.

[28] Llompart-Pou JA, Barea-Mendoza JA, Sanchez-Casado M, Gonzalez-Robledo J, Mayor-Garcia DM, Montserrat-Ortiz N (2020). Neuromonitoring in the severe traumatic brain injury. Spanish Trauma ICU Registry (RETRAUCI). Neurocirugia (Astur).

