# Supplementary material for: Characteristics and therapeutic profile of TBI patients who underwent bilateral decompressive craniectomy: experience with 151 cases
Source: Scand J Trauma Resusc Emerg Med. 2022 Nov 17;30:59. doi: 10.1186/s13049-022-01046-w (PMC9670501; doi:10.1186/s13049-022-01046-w)
Supplement: Supplementary file 1 — Additional file 1: Table S1. Multivariate logistic regression analysis predicting USSs. [file 13049_2022_1046_MOESM1_ESM.docx]

Table S1. Multivariate logistic regression analysis predicting USSs

| Independent variable | Adjusted OR (95% CI) | P value |
| --- | --- | --- |
| Age | 1.04 (0.69-1.56) | 0.512 |
| GCS | 0.87 (0.58-1.67) | 0.267 |
| Motor vehicle accidents | 1.45 (0.79-1.76) | 0.103 |
| IPH | 1.13 (0.67-1.87) | 0.133 |
| Pupillary reactions | 0.81 (0.56-1.44) | 0.078 |
| Coagulopathy | 1.58 (0.93-2.03) | 0.087 |
| NLR | 1.08 (0.71-1.59) | 0.225 |

ICP: Intracranial Pressure; IPH: intra-parenchyma hematoma; NLR: Neutrophil-to-Lymphocyte Ratio; USS: Unplanned secondary surgery.
