# Supplementary material for: Characteristics and therapeutic profile of TBI patients who underwent bilateral decompressive craniectomy: experience with 151 cases
Source: Scand J Trauma Resusc Emerg Med. 2022 Nov 17;30:59. doi: 10.1186/s13049-022-01046-w (PMC9670501; doi:10.1186/s13049-022-01046-w)
Supplement: Supplementary file 2 — Additional file 1: Table S2. 3-month complications comparison between USS and none-USS patients. [file 13049_2022_1046_MOESM2_ESM.docx]

Table S1. 3-month complications comparison between USS and none-USS patients

|  | USS patients, n=44 | None-USS patients, n=107 | P value |
| --- | --- | --- | --- |
| Hydrocephalus | 18 (40.9) | 22 (20.6) | 0.0145* |
| CNS Infection | 5 (11.4) | 12 (11.2) | > 0.99 |
| Seizure attacking | 16 (36.3) | 14 (13.1) | 0.0028* |
| Subdural hygroma | 17 (38.6) | 28 (26.2) | 0.170 |
| Hypostatic pneumonia | 11 (25.0) | 32 (29.9) | 0.556 |
| Others | 12 (27.3) | 19 (17.7) | 0.192 |

Data are given as n (%) unless otherwise noted. *: p < 0.05

USS= Unplanned secondary surgery
